# B1a B cells require autophagy for metabolic homeostasis and self-renewal

**DOI:** 10.1084/jem.20170771

**Published:** 2018-02-05

**Authors:** Alexander J. Clarke, Thomas Riffelmacher, Daniel Braas, Richard J. Cornall, Anna Katharina Simon

**Affiliations:** 1 Kennedy Institute of Rheumatology and Medical Research Council Human Immunology Unit, Weatherall Institute of Molecular Medicine, University of Oxford, Oxford, England, UK; 2 Nuffield Department of Medicine, Medical Research Council Human Immunology Unit, Weatherall Institute of Molecular Medicine, University of Oxford, Oxford, England, UK; 3 Department of Molecular and Medical Pharmacology and UCLA Metabolomics Center, University of California, Los Angeles, Los Angeles, CA

## Abstract

Specific metabolic programs are activated by immune cells to fulfill their functional roles, which include adaptations to their microenvironment. B1 B cells are tissue-resident, innate-like B cells. They have many distinct properties, such as the capacity to self-renew and the ability to rapidly respond to a limited repertoire of epitopes. The metabolic pathways that support these functions are unknown. We show that B1 B cells are bioenergetically more active than B2 B cells, with higher rates of glycolysis and oxidative phosphorylation, and depend on glycolysis. They acquire exogenous fatty acids and store lipids in droplet form. Autophagy is differentially activated in B1a B cells, and deletion of the autophagy gene *Atg7* leads to a selective loss of B1a B cells caused by a failure of self-renewal. Autophagy-deficient B1a B cells down-regulate critical metabolic genes and accumulate dysfunctional mitochondria. B1 B cells, therefore, have evolved a distinct metabolism adapted to their residence and specific functional properties.

## Abstract (precis)

Clarke et al. demonstrate that the innate-like B1 B cell subset has a distinct metabolic phenotype, characterized by high levels of glycolysis, pentose phosphate pathway, and TCA cycle activity, and depends on autophagy for metabolic homeostasis and self-renewal.

## Introduction

B1 B cells are a distinct lineage of tissue-resident, innate-like B cells with critical roles in the immune response to pathogens with repetitive carbohydrate epitopes, such as *Streptococcus pneumoniae* (Baumgarth, 2011). They are a major source of natural IgM, which, in addition to its antimicrobial properties, helps maintain tissue homeostasis by cross-reaction with epitopes expressed on dead and dying cells (Chen et al., 2009). They are also an important component of barrier immunity, as they preferentially class switch to IgA to control microbes at mucosal surfaces (Kaminski and Stavnezer, 2006). B1 B cells are normally resident in the peritoneum and pleura, although they also recirculate through secondary lymphoid tissues (Ansel et al., 2002). After activation, they transit to the spleen or draining lymph nodes, where they secrete antibodies (Yang et al., 2007). These responses are typically antigen nonspecific, as B1 B cells preferentially respond to Toll-like receptor rather than BCR signaling (Baumgarth, 2011).

B1 B cells develop distinct from B2 cells (which include follicular and marginal zone B cells), and their developmental origins have been the subject of considerable debate (Montecino-Rodriguez and Dorshkind, 2012). B1 B cells are initially seeded after generation during fetal and early neonatal life, and the major population thereafter is maintained by self-renewal (Hayakawa et al., 1986; Krop et al., 1996). B2 B cells, however, are continuously produced in the bone marrow from hematopoietic stem cells (HSCs) throughout life, although there remains limited potential for B1 production from bone marrow B1 progenitors (Barber et al., 2011). B1 B cell selection is enhanced by strong BCR signaling, which may be spontaneous or induced by self-antigens, and it has been proposed that this leads to their formation from a progenitor in common with B2 cells (the selection model). The alternative lineage theory is that B1 cells arise from a distinct progenitor (Tung et al., 2006). B1 B cells are recognized as CD19^hi^B220^lo^IgM^hi^CD23^−^; the major B1a subset is CD5^+^, and the minor B1b subset is CD5^−^. B1b B cells recognize a broader range of antigens and can form memory B cells (Baumgarth, 2011).

It has become established that T lymphocytes adopt distinct metabolic programs that are highly regulated between functional subsets. Naive T cells mainly generate energy by mitochondrial oxidative phosphorylation (OXPHOS). Upon activation, T cells additionally up-regulate aerobic glycolysis; that is, a reduction of pyruvate produced by glycolysis to lactate (Buck et al., 2015). OXPHOS is then down-regulated as the T cell becomes a fully differentiated effector. Regulatory T cells, in comparison, predominantly generate energy by fatty acid oxidation (Michalek et al., 2011), as do memory T cells, which is thought to reflect their residence in lipid-rich microenvironments such as the skin, lymph node, and intestinal lamina propria (Pearce et al., 2009; Pan et al., 2017). Innate lymphoid cells have also recently been shown to predominantly use environmental fatty acids (Wilhelm et al., 2016).

In contrast, comparatively little is known about the metabolic phenotypes of nonmalignant B cells, and, in particular, the metabolic programs that maintain B cell homeostasis in vivo have been much less explored (Pearce and Pearce, 2013). The distinct tissue residence of B1a B cells in the peritoneum, which is a highly lipid-rich environment, coupled with their self-renewal capacity and state of preactivation suggests that they may have evolved a specific metabolic program to support these characteristics. Importantly, chronic lymphoid leukemia is thought to frequently arise from B1 B cells, and therefore understanding their underlying metabolism may lead to new therapeutic insights (Montecino-Rodriguez and Dorshkind, 2012).

Here, we show that B1a B cells engage a metabolic program distinct from follicular B2 (Fo B2) B cells. They have active glycolysis and fatty acid synthesis, with little metabolic flexibility. They acquire exogenous lipids and maintain intracellular fat stores. They are dependent, unlike Fo B2 B cells, on autophagy to survive and self-renew, and loss of autophagy causes global metabolic dysfunction and failure of lipid and mitochondrial homeostasis.

## Results and discussion

### B1a B cells have a distinct metabolic gene transcription identity

**Figure 1.**
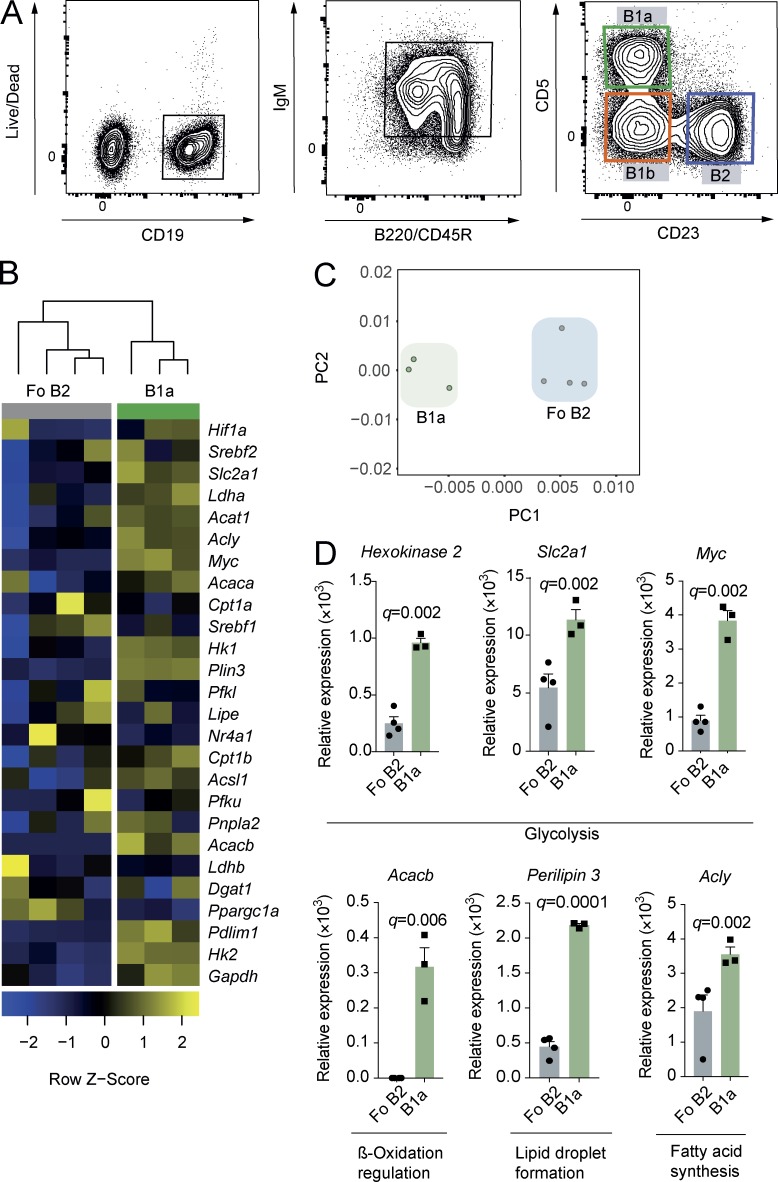
**B1a B cells have a distinct metabolic gene transcription identity. (A)** Gating strategy for the identification of mouse peritoneal B cell subsets. B1a B cells were identified as viable, CD19^+^, IgM^hi^, B220^lo^, CD23^−^, or CD5^+^. B2 B cells were CD23^+^CD5^−^. B1b B cells were CD23^−^CD5^−^. **(B)** Heat map of Fluidigm Biomark qRT-PCR gene expression data for peritoneal B1a B cells (CD19^+^C23^−^CD5^+^) and splenic Fo B2 B cells (CD19^+^CD23^+^). 1,000 cells were sorted by flow cytometry according to the gating strategy in A, and qRT-PCR was performed for a curated metabolic gene set (Table S1). Data are target gene expression relative to *β-actin*, and heat map coloring is based on row Z score. Each data point represents one C57BL/6 WT mouse and is the mean of two technical replicates. Hierarchical clustering is unsupervised. **(C)** Principal component analysis of data from B. First and second principal components are plotted. **(D)** Gene expression data from B. Each data point is one mouse, with two technical replicates each. Unpaired Student’s *t* test p-value is adjusted for multiple testing (q-value; using FDR method; 5% threshold). The post-hoc p-value for *Acly* is presented unadjusted. Each significantly differentially expressed gene was independently confirmed by qRT-PCR. Mean ± SEM is depicted.

To determine whether differences exist in the expression of key metabolic genes between CD5^+^CD23^−^ peritoneal B1a and splenic CD23^+^ Fo B2 B cells, we performed multiplex quantitative real-time PCR (qRT-PCR) using Fluidigm Biomark on cells sorted by flow cytometry (Fig. 1 A). Principal component analysis revealed clear separation between the cell types, as did unsupervised hierarchical clustering (Fig. 1, B and C). We found significantly higher expression of genes critical for glucose uptake and commitment to glycolysis (*Slc2a1* [Glut1], *Hk2* [Hexokinase 2], and *Myc* [c-Myc]), regulation of fatty acid synthesis (*Acacb* [Acetyl-CoA carboxylase 2]), and lipid droplet formation (*Plin3* [Perilipin-3]) in B1a B cells compared with Fo B2 B cells (Fig. 1 D). Post-hoc analysis of other fatty acid metabolic genes also showed up-regulation of *Acly* (ATP citrate lyase, which catalyzes the formation of acetyl-CoA from citrate for fatty acid synthesis; Fig. 1 D). Gene expression data were therefore suggestive of a distinct metabolic program in B1a B cells compared with Fo B2 B cells, characterized by high expression of glycolysis genes, but also those important in fatty acid metabolism, synthesis, and storage.

### B1 B cells have high levels of glycolysis and OXPHOS

**Figure 2.**
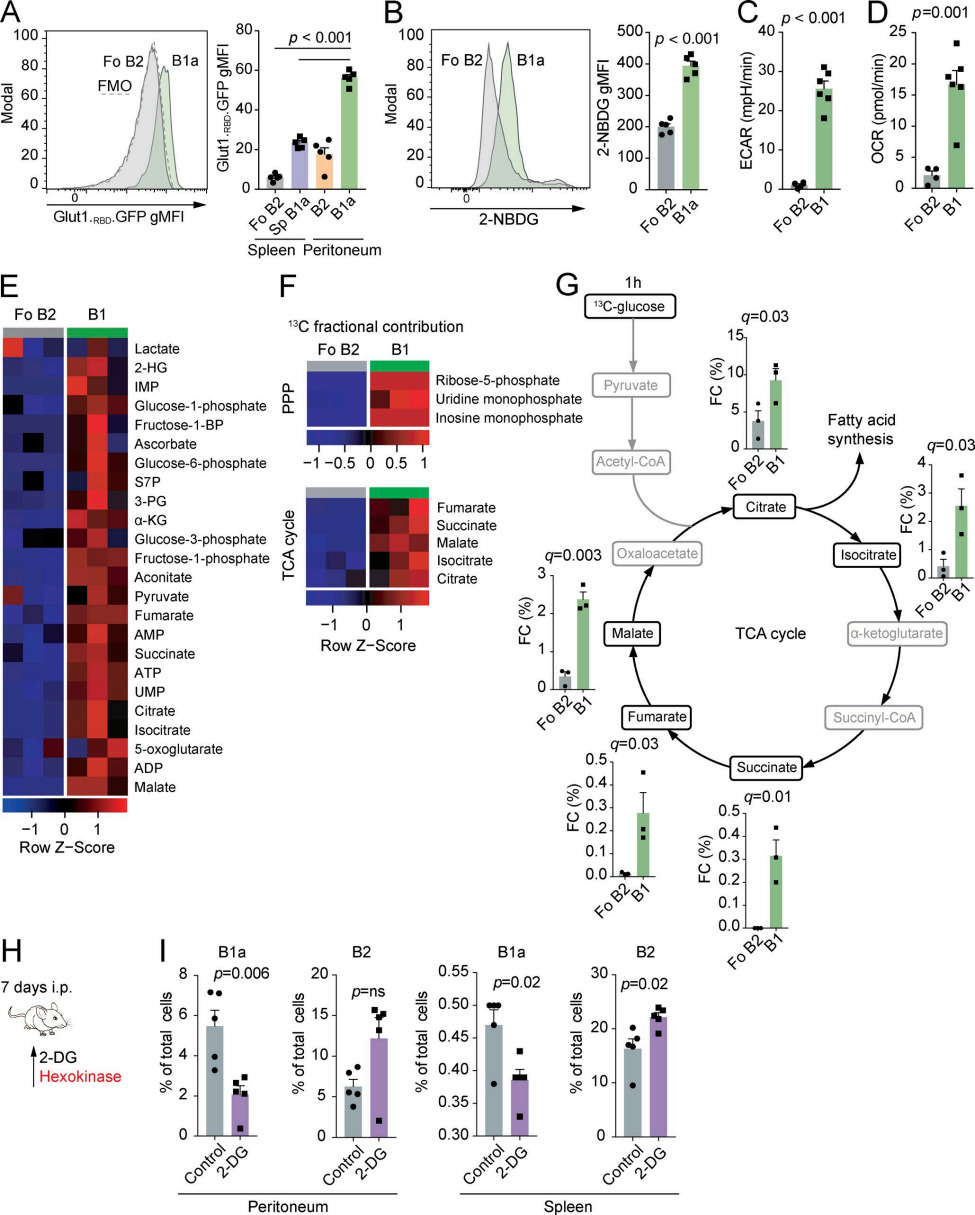
**B1a B cells have active glycolysis and OXPHOS and low metabolic flexibility in vivo. (A)** Representative histogram of Glut1._RBD_.GFP staining in peritoneal B1a and Fo B2 B cells and quantification of geometric mean fluorescence intensity (gMFI) of Glut1._RBD_.GFP staining in B1a and B2 cells from the spleen and peritoneum. *n* = 5 WT C57BL/6 individual mice per group. One-way ANOVA with Dunnett correction for multiple testing used. FMO, fluorescence minus one. **(B)** Representative histogram of 2-NBDG uptake in peritoneal B1a and Fo B2 B cells and gMFI of 2-NBDG in splenic Fo B2 cells and peritoneal B1a B cells. *n* = 5 WT C57BL/6 individual mice per group. Unpaired Student’s *t* test used. **(C)** Basal extracellular acidification rate (ECAR) of peritoneal total B1 B cells (B1a and B1b) compared with Fo B2 cells. Data are *n* = 5 technical replicates of cells sorted from pools of 10 WT C57BL/6 mice. Unpaired Student’s *t* test used. **(D)** Basal oxygen consumption rate (OCR) of peritoneal total B1 B cells (B1a and B1b) compared with Fo B2 B cells. Data are *n* = 5 technical replicates of cells sorted from pools of 10 WT C57BL/6 mice. Unpaired Student’s *t* test used. **(E)** Heat map of relative abundance of polar metabolites extracted from peritoneal total B1 (CD19^+^CD23^−^) and splenic Fo B2 B cells (CD19^+^CD23^+^) sorted by flow cytometry. Each data point represents cells sorted from pools of five to seven 6-wk-old WT C57BL/6 mice. Heat map coloring is based on row Z score. 2-HG, 2-hydroxyglutarate; IMP, inosine monophosphate; S7P, sedoheptulose-7-phosphate; 3-PG, 3-phosphoglycerate; α-KG, α-ketoglutarate; UMP, uracil monophosphate. **(F)** Heat map of fractional contribution of ^13^C to metabolites in the PPP and the TCA cycle after incubation of cells described in E with [U-^13^C]glucose for 1 h. Heat map coloring is based on row Z score. **(G)** Fractional contribution (FC) of ^13^C to metabolites in the TCA cycle after incubation of cells described in E with [U-^13^C-glucose] for 1 h. Unpaired Student’s *t* test p-value is adjusted for multiple testing using FDR method (q-value; 5% threshold). (A–G) Data representative of two independent experiments. **(H)** Experimental schematic. WT C57BL/6 mice were injected i.p. with 2-DG or PBS (control) each day for 7 d. The mechanistic target of 2-DG is illustrated in red. **(I)** Effect of 2-DG or PBS on relative cellularity of indicated B cell subset at specified location. Each data point represents an individual mouse. *n* = 5 biological replicates. P-values generated by ANOVA are relative to control, with Dunnett correction for multiple comparisons. Representative of three independent experiments. Mean ± SEM is depicted.

To confirm that enhanced expression of glycolysis genes had functional effects in B1a B cells, we measured glucose uptake and utilization ex vivo. Transport of glucose through Glut1 is a key regulator of glycolytic rate and has been shown to be critical in T cell homeostasis (Macintyre et al., 2014). To quantify the expression of this transporter, we measured the binding of an enhanced GFP–tagged HTLV-1 fusion protein, which specifically binds to Glut1 (Manel et al., 2003). We found that B1a B cells have increased surface levels of the Glut1 transporter compared with Fo B2 and CD23^+^ peritoneal B2 cells, which do not express CD11b and transit the peritoneum, and splenic B1a B cells, which share the same microenvironment as Fo B2 B cells (Fig. 2 A). To exclude the possibility that the differences we observed were simply the result of unequal cell size, we analyzed enhanced GFP fluorescence in peritoneal B1a and Fo B2 B cells gated to equalize flow cytometric forward scatter. Significant differences between the cell types remained (Fig. S1 A). We next quantified glucose uptake using the fluorescent d-glucose analogue 2-NBDG. 2-NBDG was taken up more avidly in B1a B cells than Fo B2 B cells (Fig. 2 B). Finally, we directly measured the basal extracellular acidification rate, which approximates to glycolytic flux, using Seahorse (Fig. 2 C). This confirmed high levels of aerobic glycolysis in B1 cells compared with Fo B2 B cells. The second major source of ATP generation is by mitochondrial OXPHOS. We also noted elevated rates of basal oxygen consumption, reflective of active OXPHOS in B1 cells (Fig. 2 D).

Having demonstrated higher glucose uptake in B1a B cells in association with increased OXPHOS, we next traced carbon distribution from glucose using uniformly labeled [^13^C]glucose (i.e., all carbons are of the ^13^C isotope), followed by ion chromatography–mass spectrometry metabolomics. We incubated ex vivo peritoneal total B1 (CD19^+^CD23^−^) and Fo B2 B cells in ^13^C-glucose–supplemented media for 1 h without stimulation to maximize fidelity to in vivo metabolism.

We found that B1 B cells had elevated total levels of glycolytic, pentose phosphate pathway (PPP), and TCA cycle intermediates compared with Fo B2 B cells (Fig. 2 E). The PPP runs parallel to glycolysis and has anabolic functions, including the provision of ribose-5-phosphate for nucleic acid synthesis and nicotinamide adenine dinucleotide phosphate for reductive reactions such as fatty acid synthesis. There was substantially more glucose-derived ^13^C contributing to the TCA cycle and PPP metabolites in B1 B cells than in Fo B2 B cells (Fig. 2 F).

Having demonstrated high levels of glycolysis and TCA cycle activity, we next determined the importance of glycolysis for B1 B cell homoeostasis in vivo. To do so, we treated mice with the compound 2-deoxyglucose (2-DG), which competitively inhibits glycolysis (Fig. 2, H and I). We found that 7 d of 2-DG treatment selectively depleted B1a B cells in both the peritoneum and spleen. 2-DG induced primary necrosis of B cells in the peritoneum, as determined by increased late necrotic cell numbers, but a reduction in caspase-3 activation (Fig. S1, B and C). Apoptosis was, however, activated in splenic B1a B cells (Fig. S1 B). There was no effect of 2-DG on levels of the cell proliferation marker Ki67 in peritoneal B1a B cells, but a decrease was seen in splenic Fo B2 B cells (Fig. S1 D). The lack of Ki67 up-regulation in peritoneal B1a B cells suggested a failure to undergo homeostatic proliferation in the face of niche depletion.

To understand whether stimulation might influence relative pathway dependence and to confirm our in vivo observations, we cultured B1 or Fo B2 B cells for 24 h in the presence or absence of the TLR9 agonist ODN1826, with or without 2-DG. We found that 2-DG increased cell death twofold in B1 B cells compared with Fo B2 B cells (Fig. S1 E). IgM production was severely reduced in both cell types (Fig. S1 F).

This result demonstrates that B1a B cells are dependent on glycolysis in vivo. Our finding that the protooncogene c-Myc is highly expressed in B1a B cells (Fig. 1 D), also recently reported by Hayakawa et al. (2016), suggests a driving mechanism for this pattern of energy metabolism, as c-Myc is a positive regulator of both glycolysis and OXPHOS (Caro-Maldonado et al., 2014). Notably, mice overexpressing c-Myc in B cells have increased B1 B cell numbers (Khuda et al., 2008).

### B1 B cells take up exogenous lipids and undergo cell death in response to inhibition of fatty acid synthesis

The finding of high levels of glucose uptake and entry into the TCA cycle and PPP was suggestive of fatty acid synthesis, and, given the residence of B1a B cells in the highly lipid-rich peritoneal microenvironment, we next considered whether B1a B cells might also acquire exogenous lipids.

**Figure 3.**
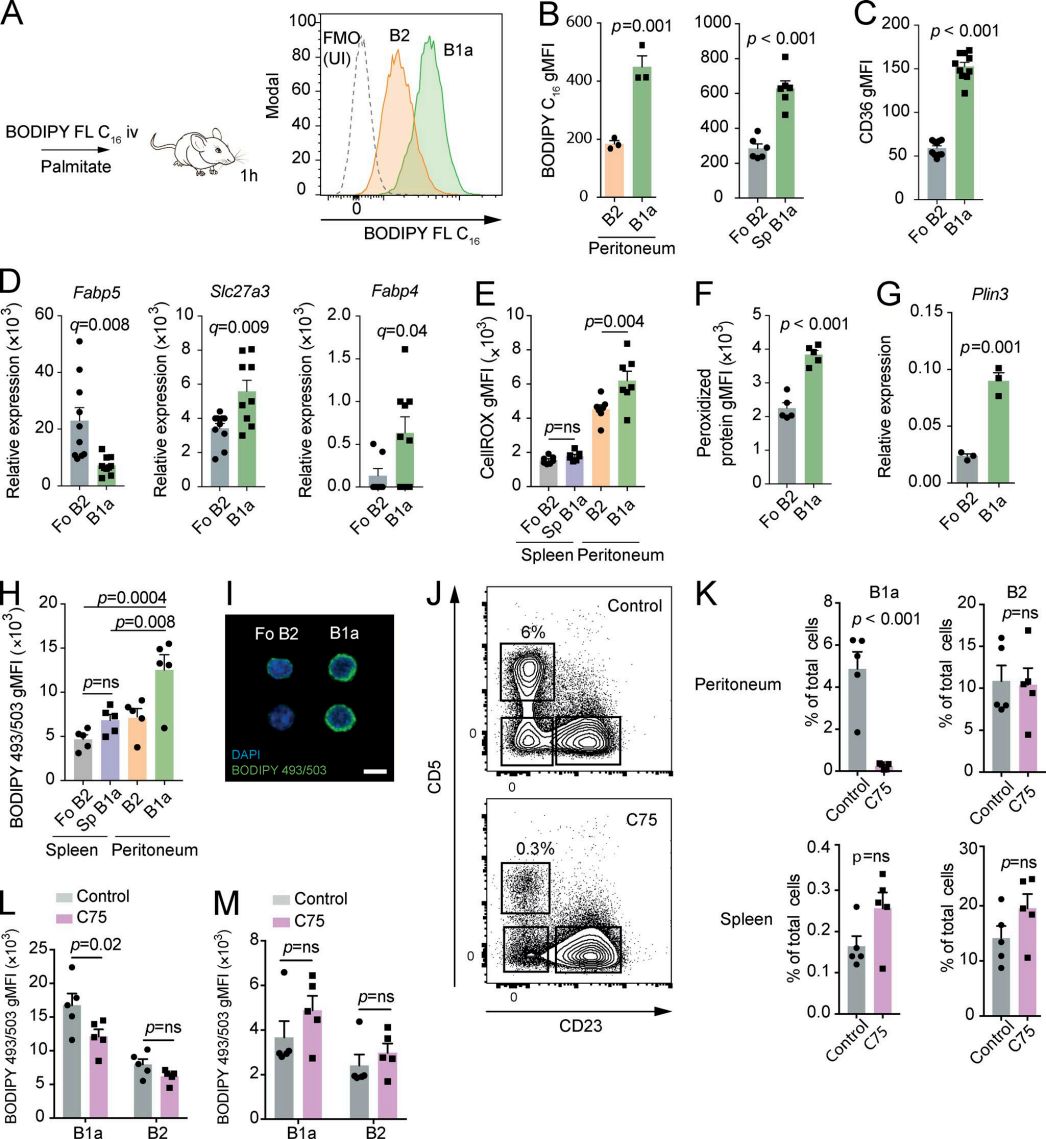
**B1 B cells take up exogenous lipids and undergo cell death in response to inhibition of fatty acid synthesis. (A)** Experimental schematic and example histograms. 6-wk-old C57BL/6 mice were injected i.v. with BODIPY FL C_16_ and then analyzed after 1 h. Example distributions of fluorescence from peritoneal B1a and B2 B cells analyzed by flow cytometry compared with an uninjected (UI) control. FMO, fluorescence minus one. **(B)** gMFI of BODIPY FL C_16_ from peritoneal B cells after injection. *n* = 3 biological replicates representative of or pooled from two independent experiments. Unpaired Student’s *t* test used. **(C)** gMFI of CD36 on peritoneal B1a and splenic Fo B2 B cells from 6-wk-old C57BL/6 mice. Each point represents a single mouse, and data are pooled from two independent experiments. Unpaired Student’s *t* test used. **(D)** Peritoneal B1a and splenic Fo B2 B cells were sorted by flow cytometry from 6-wk-old C57BL/6 mice, and qRT-PCR was performed for the indicated genes, relative to *B2m*. Each point represents a single mouse, and data are pooled from two independent experiments. Unpaired Student’s *t* test p-value is adjusted for multiple testing using FDR method (q-value; 5% threshold). **(E)** gMFI of CellROX from splenic and peritoneal B cells of 6-wk-old C57BL/6 mice. *n* = 5 biological replicates. One-way ANOVA with Dunnett correction for multiple comparisons used. *n* = 5 biological replicates. Representative of two independent experiments. **(F)** gMFI of Alexa Fluor 488–ClickIT lipid peroxidation assay for peritoneal B1a and splenic Fo B2 B cells of 6-wk-old C57BL/6 mice. *n* = 5 biological replicates. Unpaired Student’s *t* test used. Representative of two independent experiments. **(G)** Expression of *Plin3* relative to *B2m* in flow-sorted peritoneal B1a and splenic Fo B2 B cells from 6-wk-old C57BL/6 mice. *n* = 3 biological replicates. Unpaired Student’s *t* test used. Representative of two independent experiments. **(H)** gMFI of BODIPY 493/503 in splenic Fo B2 B cells and peritoneal B1a and B2 B cells of 6-wk-old C57BL/6 mice. *n* = 5 biological replicates, pooled from two independent experiments. One-way ANOVA with Dunnett correction for multiple comparisons used. **(I)** Representative microscope images of BODIPY 493/503 staining in flow-sorted peritoneal B1a and splenic Fo B2 B cells in 6-wk-old C57BL/6 mice. Bar, 20 µm. Representative of two independent experiments. **(J–M)** 6-wk-old C57BL/6 mice received i.p*.* injections of C75 (15 mg⋅kg^−1^) every 2 d for three doses before sacrifice. Shown are representative flow cytometry plots of peritoneal CD19^+^ B cells from C75 injected and control mice (H). Shown is the percentage of the indicated population of total cells in the analyzed compartment (I) or the gMFI of BODIPY 493/503 measured by flow cytometry (J and K). Each point represents one mouse. Unpaired Student’s *t* test used in I, two-way ANOVA with Sidak correction for multiple testing used in J and K. Representative of three independent experiments. Mean ± SEM is depicted. ns, not significant.

We injected mice i.v. with a fluorescently labeled form of the long-chain fatty acid palmitate (BODIPY FL C_16_) and analyzed them after 1 h (Fig. 3, A and B). We found that peritoneal B1a B cells have increased fatty acid uptake compared with peritoneal CD23^+^ B2 cells, a finding preserved in the spleen, suggesting that a high capacity to take up free fatty acids is a persistent attribute of B1a B cells regardless of their local environment.

To analyze how these lipids might be taken up, we next quantified surface expression of the fatty acid transporter protein CD36 by flow cytometry. We found approximately threefold higher levels of CD36 on peritoneal B1a B cells compared with Fo B2 B cells (Fig. 3 C). We also examined expression of other lipid transporter genes by qRT-PCR and found higher levels of *Slc27a3* and *Fabp4* transcription in B1a B cells, but decreased *Fabp5* expression compared with Fo B2 B cells, suggesting subset-specific lipid transporter use (Fig. 3 D).

The presence of high concentrations of intracellular free fatty acids can lead to oxidative stress caused by lipid peroxidation (Hauck and Bernlohr, 2016). In keeping with this, we found elevated levels of cellular ROS and lipid peroxidation–derived protein modifications in B cells from the peritoneum compared with the spleen and a relative increase in these parameters in the peritoneal B1a compared with B2 populations (Fig. 3, E and F), suggesting that the peritoneal microenvironment inherently induces oxidative stress in association with lipid flux. To mitigate lipotoxicity, triglycerides are synthesized from fatty-acyl CoA, which are then stored in lipid droplets (Wilfling et al., 2014). The finding that expression of the lipid droplet–associated gene *Perilipin-3* is increased in B1a B cells suggests that they may store excess lipid in droplet form (Fig. 3 G). To explore this, we measured neutral lipid storage by staining with the BODIPY 493/503 tracer molecule. By flow cytometry and microscopy, we found significantly more lipid droplets in B1a B cells compared with Fo B2 B cells (Fig. 3, H and I).

To directly assess the importance of fatty acid synthesis to the maintenance of B1a B cells, we treated mice with three doses of the fatty acid synthase inhibitor C75 on alternate days. C75 treatment led to a dramatic depletion of peritoneal B1a B cells, with no significant effect on peritoneal B2 B cells or on either subset in the spleen (Fig. 3, J and K). In keeping with its mode of action, neutral lipid stores were depleted in peritoneal B1a B cells, but not in peritoneal B2 B cells or in splenic populations (Fig. 3, L and M). The lack of impact on B2 B cells is in accordance with previous work in vitro, which showed no effect of C75 on splenic B cells (Bhatt et al., 2012). To analyze how C75 might lead to B1 B cell depletion, we analyzed caspase-3 activation and levels of the proliferation marker Ki67 by intracellular flow cytometry after the three-dose regimen. We found that Ki67 levels were lower in the splenic B1a B cells of C75-treated mice, but no differences were observed in peritoneal B cell subsets or in caspase-3 activation (Fig. S2, A and B). This suggests that in the peritoneum, B1 cell loss occurs rapidly and that the expected increase in Ki67 as B1a B cells expand to fill the deficient niche does not occur.

These results together indicate that B1a B cells both acquire exogenous lipids and also require fatty acids synthesized de novo, which is likely to be from citrate produced as a product of glycolysis, through the enzyme ATP-citrate lyase.

### Autophagy is required for B1 B cell survival and self-renewal

Autophagy is a highly conserved process for the degradation of cellular macromolecules and organelles by the lysosome (Levine et al., 2011). This pathway is therefore capable of supplying critical substrates to fuel metabolism (Kaur and Debnath, 2015; Riffelmacher et al., 2017).

Autophagy has been shown to be required for the control of cellular lipid dynamics, with roles both in degradation and lipid droplet formation (Singh et al., 2009; Liu and Czaja, 2013; Rambold et al., 2015). Active autophagy is a key attribute maintaining the metabolic state required for self-renewal (Mortensen et al., 2011; Guan et al., 2013; Pan et al., 2013; García-Prat et al., 2016). In the resting state, deletion of autophagy genes has been reported to selectively lead to loss of B1 B cells (Miller et al., 2008). We reasoned that autophagy may therefore be critical in maintaining normal B1 B cell metabolic homeostasis by the provision of metabolites such as free fatty acids, which would also therefore affect their self-renewal capacity.

**Figure 4.**
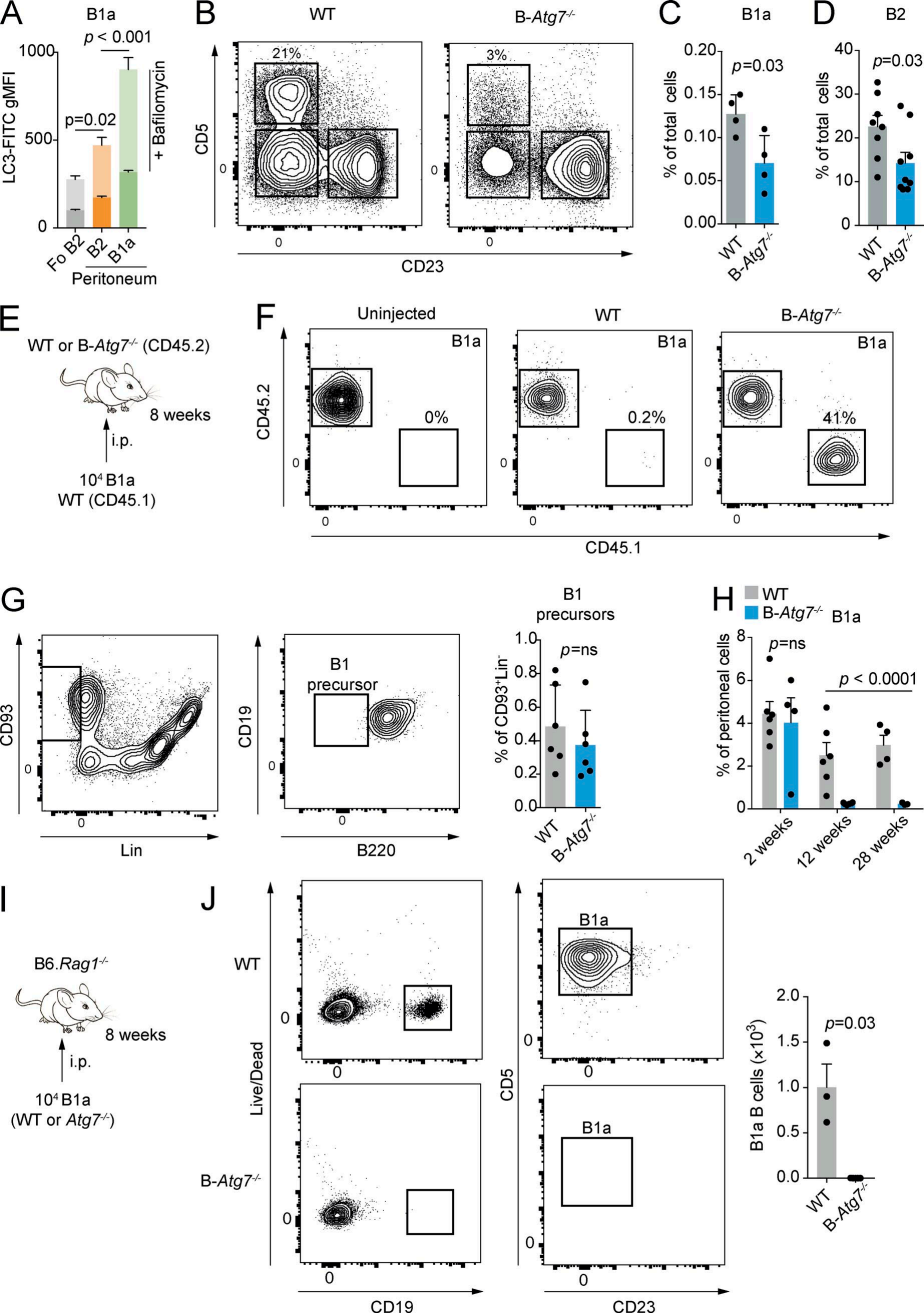
**Autophagy is required for B1a survival and self-renewal. (A)** gMFI of LC3-FITC in splenic Fo B2 and peritoneal B1a and B2 B cells after washout of LC3-I. Overlay bars denote gMFI after incubation with 10 nM bafilomycin A_1_ for 30 min. *n* = 5 biological replicates pooled from two independent experiments. One-way ANOVA with Dunnett correction for multiple testing calculated on postbafilomycin values. **(B)** Representative example of peritoneal CD19^+^ B cell compartment of control and B-*Atg7*^−/−^ mice. **(C)** Splenic B1a B cells as a percentage of total splenic cells in control and B-*Atg7^−/−^* mice. Each point represents one mouse. Unpaired Student’s *t* test used. Representative of at least four independent experiments. **(D)** Peritoneal B2 B cells as a percentage of total peritoneal cells in control and B-*Atg7^−/−^* mice. Each point represents one mouse. Unpaired Student’s *t* test used. Representative of more than four independent experiments. **(E)** Experimental schematic. 10^4^ sorted peritoneal B1a B cells from CD45.1 WT mice were transferred by i.p. injection to either control or B-*Atg7^−/−^* mice (both CD45.2). Representative of two independent experiments. **(F)** Representative flow cytometric plot of peritoneal cells from E. **(G)** Gating strategy for bone marrow B1 precursors. Cells were defined as CD93^+^Lin^−^CD19^+^B220^−^. B1 precursor cells as a percentage of total bone marrow cells in control and B-*Atg7^−/−^* mice are shown. Each point represents one mouse. Representative of two independent experiments. ns, not significant. **(H)** Peritoneal B1a B cells as a percentage of total peritoneal cells in control and B-*Atg7^−/−^* mice at 2, 12, and 28 wk of age. Each point represents one mouse. Data pooled from two independent experiments. Two-way ANOVA with Sidak correction for multiple comparisons used. **(I)** Experimental schematic. 10^4^ sorted peritoneal B1a B cells from control and B-*Atg7^−/−^* mice were adoptively transferred by i.p. injection to B6.*Rag1^−/−^* hosts and analyzed after 8 wk. **(J)** Representative flow cytometric plot of peritoneal cells from I. Each point represents one mouse. Unpaired Student’s *t* test used. Representative of two independent experiments. Mean ± SEM is depicted.

We first measured autophagic flux by washing out nonautophagosomal LC3-I with saponin and then using immunofluorescence to detect LC3-II by flow cytometry (Fig. 4 A; Eng et al., 2010). This assay was performed with and without bafilomycin A_1_, a lysosomal V-ATPase inhibitor that prevents autophagosome–lysosome fusion and therefore reveals autophagic flux. We found considerably increased autophagy in peritoneal B1a B cells compared with Fo B2 B cells. We next conditionally deleted *Atg7* in B cells using the *Mb1*-Cre system (hereafter referred to as B-*Atg7^−/−^*; Hobeika et al., 2006). In keeping with other reports of B cell autophagy deletion at later points in B cell development, we found an essentially normal peripheral B cell compartment but markedly reduced numbers of peritoneal B1 B cells (Fig. 4 B and Fig. S3 A; Miller et al., 2008; Chen et al., 2014; Arnold et al., 2016). Also reduced were splenic B1a B cells (Fig. 4 C) and, interestingly, peritoneal B2 B cells (Fig. 4 D).

B1a cells once stimulated leave the peritoneum via the omentum and traffic to the spleen and bone marrow to produce Ig (Ansel et al., 2002; Yang et al., 2007). Because autophagy is required for plasma cell formation and maintenance (Pengo et al., 2013; Clarke et al., 2015), it was possible that B1a B cells might rapidly exit the peritoneum in an attempt to regulate natural IgM levels in a B-*Atg7^−/−^* environment. To determine whether this was the case, we next adoptively transferred WT congenically marked CD45.1 B1a B cells into the peritonea of B-*Atg7^−/−^* mice (which express the CD45.2 antigen; Fig. 4, E and F). We found that WT CD45.1 B1a B cells remained in the peritoneum and effectively self-renewed after transfer, excluding the possibility that autophagy-deficient B1a B cells were simply dispersing. Next, to determine whether the B1 B cell defect was caused by a failure of normal development, we examined the numbers of CD93^+^Lin^−^CD19^+^B220^+^ B1 precursor cells in B*-Atg7^−/−^* mice (Fig. 4 G), which are generated throughout life at a low level from the bone marrow (Esplin et al., 2009). We found no difference in these progenitors compared with control mice, suggesting that their maintenance does not require autophagy. To examine the kinetics of B1 B cell loss, we analyzed mice at 2, 12, and 28 wk of age (Fig. 4 H). We found no difference in B1a numbers at 2 wk, but by 12 wk they had drastically decreased and remained low at 28 wk, indicating that B1 B cell fetal and neonatal differentiation is normal, but self-renewal in adult mice is affected by loss of autophagy. To confirm that autophagy is required for B1a B cell self-renewal, we adoptively transferred 10^4^ sorted autophagy-deficient or control cells into the peritonea of B6.*Rag1^−/−^* hosts (Fig. 4, I and J). After 8 wk, B-*Atg7^−/−^* B1a B cells were undetectable, in contrast to control cells, which were at this point abundant. These results therefore demonstrate a cell-intrinsic defect in the self-renewal of B1a B cells when autophagy is absent.

### Autophagy maintains B1 B cell metabolic homeostasis

**Figure 5.**
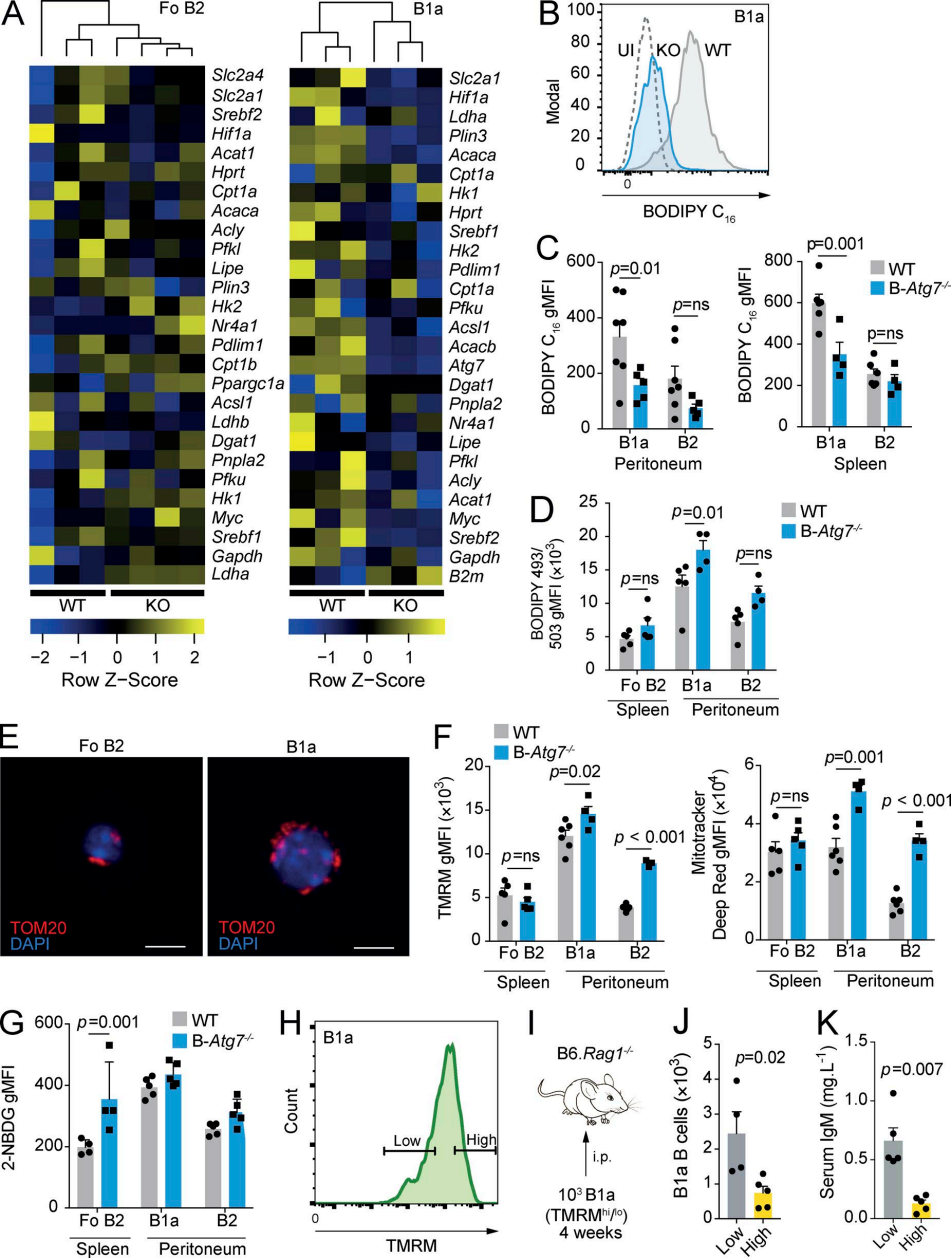
**Loss of autophagy leads to disrupted B1a B cell metabolic homeostasis. (A)** Heat map of Fluidigm Biomark qRT-PCR gene expression data for peritoneal B1a B cells and splenic Fo B2 B cells (CD19^+^CD23^+^) from control and B-*Atg7^−/−^* mice. Data are relative to *β-actin*, and coloring is based on row Z score. Each data point is the mean of three technical replicates. Hierarchical clustering is unsupervised. **(B)** Example distributions of fluorescence of BODIPY FL C_16_ from peritoneal B1a B cells from control and B-*Atg7^−/−^* mice measured by flow cytometry and compared with an uninjected (UI) WT control. **(C)** gMFI of BODIPY FL C_16_ in B1a and B2 B cells from peritoneum and spleen in control and B-*Atg7^−/−^* mice measured by flow cytometry. **(D)** gMFI of BODIPY 493/503 in B1a and B2 B cells from peritoneum and spleen in control and B-*Atg7^−/−^* mice measured by flow cytometry. (C and D) Each point represents one mouse. Data pooled from two independent experiments. Two-way ANOVA with Sidak correction for multiple testing used. **(E)** Representative immunofluorescent confocal images of the mitochondria of sorted WT Fo B2 and peritoneal B1a B cells. Cells are stained for TOM20 and nuclei are visualized with DAPI. Bars, 5 µm. **(F)** gMFI of TMRM and MitoTracker deep red in B cells from the spleen and peritoneum in control and B-*Atg7^−/−^* mice measured by flow cytometry. Each point represents one mouse. Data representative of two independent experiments. Two-way ANOVA with Sidak correction for multiple testing used. **(G)** gMFI of 2-NBDG in B1a and B2 B cells from peritoneum and spleen in control and B-*Atg7^−/−^* mice measured by flow cytometry. Each point represents one mouse. Data pooled from two independent experiments. Two-way ANOVA with Sidak correction for multiple testing used. **(H)** Flow cytometry gating definition of high and low levels of TMRM fluorescence. The highest and lowest quartiles of the distribution were used to define TMRM^hi^ and TMRM^lo^ populations of B1a B cells. **(I)** Experimental schematic. 10^3^ B1a B cells from the lowest and highest quartiles of TMRM fluorescence were sorted and adoptively transferred into the peritonea of B6.*Rag1^−/−^* hosts. These mice were then analyzed after 1 mo. **(J)** Quantification of peritoneal B1a B cells from I. **(K)** Quantification of serum IgM levels from I. (J and K) Each point represents one mouse. Unpaired Student’s *t* test used. Data representative of two independent experiments. Mean ± SEM is depicted.

To investigate the impact of autophagy deficiency on peritoneal B1a and splenic Fo B2 B cell metabolism, we repeated multiplex qRT-PCR with Fluidigm Biomark (Fig. 5 A). There was a generalized reduction in the expression of metabolism-associated genes in B1a B cells, which was much less pronounced in Fo B2 B cells. Notably down-regulated was *Acaca*, which encodes the key regulator of fatty acid synthesis, acetyl-CoA carboxylase 1. Post-hoc analysis of other lipid metabolic genes also revealed down-regulation of *Acacb*, *Acsl1*, *Plin3*, and *Srebf2* (Fig. S3 B). Expression of these genes was not affected by autophagy deficiency in Fo B2 B cells, and no other genes were significantly differentially expressed after adjustment for multiple testing. *Atg7* was effectively deleted in B-*Atg7^−/−^* B1a B cells, excluding the possibility that the residual population was caused by a failure of Cre recombinase activity (Fig. S3 C).

The down-regulation of genes involved in fatty acid synthesis by autophagy-deficient B1a B cells suggested that lipid uptake and storage might be defective. To understand how autophagy deficiency affects lipid homeostasis, we first i.v. injected control and B-*Atg7^−/−^* mice with BODIPY FL C_16_ (Fig. 5, B and C). We found that exogenous fatty acid uptake was reduced in B1a B cells from the peritoneum or spleen in B-*Atg7^−/−^* mice, but Fo B2 B cells were unaffected. However, total neutral lipid stores were in fact increased in autophagy-deficient peritoneal B1a B cells, but Fo B2 B cells were again unaffected (Fig. 5 D). These results implied that loss of autophagy inhibited fatty acid uptake in the context of accumulation of intracellular lipids and, based on transcriptional data, was associated with a decrease in lipid synthesis. The increase in lipid content of B-*Atg7^−/−^* B1a B cells can be explained by loss of lipophagy, a process by which lipid droplets are degraded by autophagy to release free fatty acids (Singh et al., 2009). The transcriptional switch away from lipid synthesis may reflect an attempt to reduce potentially toxic accumulation of intracellular lipids.

To assess to what extent mitochondrial OXPHOS might be affected by loss of autophagy, we first visualized mitochondria from Fo B2 and peritoneal B1a B cells by confocal immunofluorescent microscopy (Fig. 5 E). B1a B cells had a more extensive mitochondrial network than Fo B2 B cells, whose mitochondria were punctate. The fused mitochondrial pattern seen in B1a B cells is reported in cells with active OXPHOS, but also in those undergoing stress, which could include the lipid toxicity previously noted (Fig. 3 F; Youle and van der Bliek, 2012; Buck et al., 2016). We next quantified mitochondrial membrane potential (MMP) using tetramethylrhodamine (TMRM), a fluorescent dye that is sequestered by active mitochondria and is reflective of electron transfer chain activity. We found that in WT mice, TMRM fluorescence was significantly increased in B1a B cells compared with either Fo B2 or peritoneal B2 B cells (Fig. 5 F). However, total mitochondrial mass, measured using MitoTracker deep red, was similar in Fo B2 B cells and peritoneal B1a B cells, but much lower in peritoneal B2 B cells. These results therefore suggested that in the peritoneal microenvironment, mitochondrial activity and therefore OXPHOS is increased, which may reflect fatty acid availability as a fuel source. When we compared Fo B2 B cells of B-*Atg7^−/−^* mice with WT, we found no difference in mitochondrial mass or TMRM intensity (Fig. 5 F). Sparing of Fo B2 B cell mitochondrial function may therefore reflect either their intrinsic metabolic program or the difference in microenvironment.

We next assessed whether there was a compensatory increase in glycolysis in autophagy-deficient B cells (Fig. 5 G), as has been demonstrated in other cell types (Puleston et al., 2014; Watson et al., 2015; Kabat et al., 2016). Peritoneal B1a B cells failed to increase their uptake of 2-NBDG. However, *Atg7^−/−^* Fo B2 B cells did significantly up-regulate their glucose uptake. B1a B cells therefore have limited metabolic plasticity when autophagy is lost, unlike Fo B2 B cells, which are able to compensate by up-regulation of glycolytic activity. This lack of flexibility is in keeping with in vivo inhibitor data (Fig. 2 I). Direct measurement of metabolite levels or assessment of extracellular and metabolic flux was not possible in *Atg7^−/−^* B1a B cells because of their severely reduced numbers.

Recent work has shown that MMP is an important determinant of the capacity of several cell types, including HSCs and CD8^+^ T cells, to self-renew (Sukumar et al., 2016; Vannini et al., 2016). CD8^+^ T cells with low MMP have increased free fatty acids and lower rates of OXPHOS and an enhanced capacity to self-renew after adoptive transfer (Sukumar et al., 2016). Moreover, autophagy was found to be more active in HSCs that had undergone mitochondrial depolarization (Vannini et al., 2016). After finding that loss of autophagy in peritoneal B1a B cells resulted in an increase in MMP, we hypothesized that an increase in hyperpolarized mitochondria might mechanistically contribute to the self-renewal defect seen when autophagy is deleted. We therefore sorted WT peritoneal B1a B cells with high and low levels of MMP and adoptively transferred these subsets into B6.*Rag1^−/−^* mice (Fig. 5, H–J). There were significantly fewer B1a B cells detected in the peritonea of mice that had received cells with high MMP compared with those that had received the low MMP subset, and natural serum IgM levels were also accordingly lower (Fig. 5 K). These data therefore suggest that accumulation of active mitochondria is associated with the failure of self-renewal seen in B cells that lack autophagy.

We have demonstrated that B1a B cells engage a specific, autophagy-dependent, metabolic program to survive and self-renew in the peritoneal microenvironment, characterized by lipid uptake and predominant fatty acid synthesis, but also high levels of glycolysis, PPP, and TCA activity. The evolution of this pattern of metabolism is in keeping with the ability of B1 B cells to rapidly respond to infection, compared with B2 B cells. Interestingly, this activated state in also seen in cells that have undergone malignant transformation (Bhatt et al., 2012). Many forms of leukemia are CD5^+^, and it has been demonstrated that early generated B1-like B cells are a source of chronic lymphocytic leukemia (Hayakawa et al., 2016). Chronic lymphocytic leukemia has been found to have many metabolic features in common with our observations in B1 B cells, including high rates of lipid storage, glycolysis, and OXPHOS (Doughty et al., 2006; Tili et al., 2012; Jitschin et al., 2014; Rozovski et al., 2016).

Understanding whether the physiological metabolic phenotype we have described in B1 B cells contributes to their malignant potential remains an area for further study, as does the potential for inhibition of fatty acid synthesis or autophagy as a novel therapeutic target.

## Materials and methods

### Mice

*Atg7* was conditionally deleted in B cells by the generation of *Mb1*-cre × *Atg7^F/F^* mice (Hobeika et al., 2006). Littermate controls were used in comparative experiments. CD45.1 B6.SJL and C57BL/6 mice were purchased from Harlan and acclimatized before use. B6.*Rag1^−/−^* were supplied by F. Powrie (University of Oxford, Oxford, England, UK) and bred in the local animal facility. All mice were housed in a specific pathogen-free environment and used from between 6 and 12 wk of age unless otherwise indicated. Male and female mice were used equally. Animal experiments were approved by the local ethical review committee and performed under UK Project License PPL 30/3388.

### Cell isolation and flow cytometry

Peritoneal cells were extracted by lavage. Splenic cells were prepared by straining through a 70-µm mesh, and then red blood cells were lysed with red cell lysis buffer (Biolegend). For flow cytometry, dead cells were excluded using fixable viability dye 780 (eBioscience), and Fc receptors were blocked with anti-CD16/32 antibody (93; Biolegend). Fluorochrome-conjugated anti-CD19 (6D5), anti-CD5 (53-7.3), anti-CD23 (B3B4), anti-IgM (RMM-1), anti-B220 (RA3-6B2), anti-CD45.1 (A20), anti-CD45.2 (104), anti-CD93 (AA4.1; eBioscience), antilineage cocktail (eBioscience), anti-CD21 (7E9), and anti-CD36 (HM36) antibodies were used for surface staining (all from Biolegend unless otherwise specified). Flow cytometry was performed on LSR II or Fortessa X20 instruments (BD Biosciences). Flow sorting was performed on a flow cytometer (FACSAria III; BD Biosciences). Intracellular staining for anti–active caspase-3 (C92-605; BD Biosciences) and Ki67 (11F6; Biolegend) was performed after fixation and permeabilization (FoxP3/transcription factor buffer set; eBioscience).

### Adoptive transfer

B1a B cells were sorted from the peritoneal lavage of donor mice by flow cytometry using the gating strategy presented in Fig. 1 A. Cells were transferred by i.p. injection.

### In vivo inhibitor treatment

Mice were treated with daily i.p. injections of 1 g⋅kg^−1^ 2-DG (Sigma) or PBS for 7 d and then sacrificed. 15 mg⋅kg^−1^ C75 (Cayman Chemical) was given three times per week for 1 wk. Doses and regimes were selected with reference to the literature (Loftus et al., 2000; Wilhelm et al., 2016).

### In vitro inhibitor treatment

Cells were cultured in RPMI 1640 medium supplemented with 10% FBS, 2 mM glutamine, and 50 µM 2-mercaptoethanol (complete RPMI) and stimulated with 0.5 µM ODN1826 (Miltenyi Biotec). 2-DG was used at 2.5 mM, selected with reference to the literature (Sukumar et al., 2013).

### BODIPY C_16_ uptake in vivo

Mice were injected i.v. with 50 µg BODIPY FL C_16_ (Life Technologies) in 100 µl PBS and then sacrificed after 1 h.

### Neutral lipid staining

Cells were stained with BODIPY 493/503 (Life Technologies) at a concentration of 1 µg⋅ml^−1^ in complete RPMI for 30 min at 37°C and then washed in PBS three times before analysis by flow cytometry or microscopy.

### Measurement of autophagy

Cells were incubated in complete RPMI for 30 min in the presence of 10 nM bafilomycin A_1_ (Sigma), and then LC3 intracellular staining was performed after permeabilization using the FlowCellect LC3 antibody–based assay kit (EMD Millipore) in accordance with the manufacturer’s instructions.

### Confocal microscopy

Cells were sorted by flow cytometry and then fixed with fixation buffer (Biolegend). They were then permeabilized with 0.1% Triton X-100 and blocked with Starting Block (Thermo Fisher). Primary antibody staining was with rabbit anti-TOM20 (Santa Cruz), and secondary Alexa Fluor 488 goat anti–rabbit antibody was used for visualization. Cells were imaged with a TE2000 microscope (Nikon). Image deconvolution was performed with ImageJ (National Institutes of Health).

### Cellular ROS and lipid peroxidation measurement

Cells were stained using CellROX green (Life Technologies) at a concentration of 5 µM at 37°C in complete RPMI for 30 min and then washed in PBS three times. For determination of lipid peroxidation, the Click-IT Lipid Peroxidation kit Alexa Fluor 488 (Life Technologies) was used as per the manufacturer’s instructions.

### Mitochondrial dye assays

For MMP, cells were incubated with TMRM (Life Technologies) at a concentration of 25 nM in complete RPMI for 30 min at 37°C and then washed in PBS three times. For mitochondrial mass, cells were incubated with MitoTracker deep red (Life Technologies) at a concentration of 100 nM in complete RPMI for 30 min at 37°C and then washed in PBS three times.

### Glucose uptake

To measure surface Glut1 expression, cells were incubated with 2.5 µl per test of Glut1._RBD_.GFP (Metafora Biosystems) for 20 min at 37°C in complete media containing 0.09% sodium azide (Sigma) and then analyzed by flow cytometry. To measure glucose uptake, cells were incubated with 10 µM 2-NBDG (Sigma) for 30 min at 37°C in complete media and then analyzed by flow cytometry.

### Fluidigm Biomark

Peritoneal B1a and Fo B2 B cells were flow sorted (200 cells per population) into OneStep lysis buffer (Invitrogen). RNA was reverse transcribed and cDNA was preamplified using the CellsDirect OneStep q-RT kit (Invitrogen). The selected metabolic genes (Table S1) were amplified and analyzed for expression using a dynamic 48 × 48 array (Biomark; Fluidigm) as previously described by Tehranchi et al. (2010). Data were analyzed using the 2^−ΔCt^ method, and all results were normalized to *β-actin*, which was selected as the optimum housekeeping gene from the panel. Any genes not detected in both comparative populations were excluded from analysis. Heat map generation, hierarchical clustering, and principal component analysis were performed in R (3.2.3) using the package gplots. Gene expression differences were calculated by Student’s *t* test, and p-values were adjusted using the method of Benjamini and Hochberg with the false discovery rate (FDR) set to 5%.

### Mass spectrometry–based metabolomics analysis using ion chromatography

3 × 10^5^ peritoneal B1 B cells (CD19^+^CD23^−^) and splenic Fo B2 B cells (CD19^+^CD23^+^) were sorted by flow cytometry. Isolated cells were incubated in RPMI (glucose-free formulation) containing 10 mM [U-^13^C]glucose (Cambridge Isotope Laboratories), 2 mM glutamine, and 10% dialyzed FBS (Thermo Fisher) at 37°C for 1 h. 3 × 10^5^ cells were washed in 150 mM of ice-cold ammonium acetate, pH 7.3, and metabolites were extracted in 80% methanol on dry ice before evaporation under vacuum. Dried metabolites were resuspended in 50 µl of 50% acrylonitrile, and 5 µl was injected for chromatographic separation using the Ion Chromatography System 5000 (Thermo Fisher) coupled to a Q Exactive run in negative polarity mode (Thermo Fisher; Nagaraj et al., 2017). The gradient ran from 5 mM to 95 mM KOH over 18 min with a flow rate of 350 µl⋅min^−1^. The settings for the HESI-II source were S-lens, 50; Sheath Gas, 18; Aux Gas, 4; spray heater, 320°C; and spray voltage, −3.2 kV. Metabolites were identified based on accurate mass (±3 ppm) and retention times of pure standards. Relative amounts, mass isotopologue distributions, and fractional contributions of metabolites were quantified using TraceFinder 3.3. Heat map generation and hierarchical clustering were performed in R (3.4.3) using the package gplots.

### Metabolic analysis with Seahorse extracellular flux analyzer

The real-time extracellular acidification rate and oxygen consumption rate were measured using an extracellular flux analyzer (XFe 96; Agilent). Cells were seeded at 2 × 10^5^ cells per well in Seahorse base medium supplemented with 10 mM glucose, 2 mM glutamine, and 1 mM sodium pyruvate. Cells were rested for 1 h at 37°C before analysis.

### ELISA

Total serum IgM was measured according to the manufacturer’s instructions (Invitrogen).

### qRT-PCR

RNA was isolated using the RNeasy Micro kit (Qiagen) and reverse transcribed using the High Capacity cDNA Reverse Transcription kit (Thermo Fisher). qRT-PCR was performed using Taqman Gene Expression Master Mix on a ViiA 7 instrument (Thermo Fisher). The PCR probes used are listed in Table S1.

### Statistics

Data were analyzed with Prism 7 (GraphPad). Two populations were compared by unpaired Student’s *t* test. Three or more sample populations were compared by one-way ANOVA with multiple testing corrections according to the method of Dunnett. Groups of populations were compared by two-way ANOVA with multiple testing corrections using the method of Sidak. P-values were considered significant if <0.05.

### Online supplemental material

Fig. S1 shows that Glut1 expression is higher in B1a B cells than in Fo B2 B cells after adjustment for size and also shows the effect of 2-DG on B cell apoptosis, cell death, and IgM secretion. Fig. S2 shows the effect of C75 on B cell apoptosis and proliferation. Fig. S3 shows B2 B cell populations in B-*Atg7^−/−^* mice, the effect of *Atg7* deletion on selected metabolic gene expression, and confirmation of efficient Cre recombinase–mediated deletion of *Atg7* in B1a B cells. Table S1 lists the primer panel used in Biomark experiments.

## Supplementary Material

Supplemental Materials (PDF)
