# Supplementary material for: B1a B cells require autophagy for metabolic homeostasis and self-renewal
Source: J Exp Med. 2018 Feb 5;215(2):399–413. doi: 10.1084/jem.20170771 (PMC5789411; doi:10.1084/jem.20170771)
Supplement: Supplemental Materials (PDF) [file JEM_20170771_sm.pdf]

Journal of Experimental Medicine

SUPPLEMENTAL MATERIAL

Clarke et al., https://doi.org/10.1084/jem.20170771

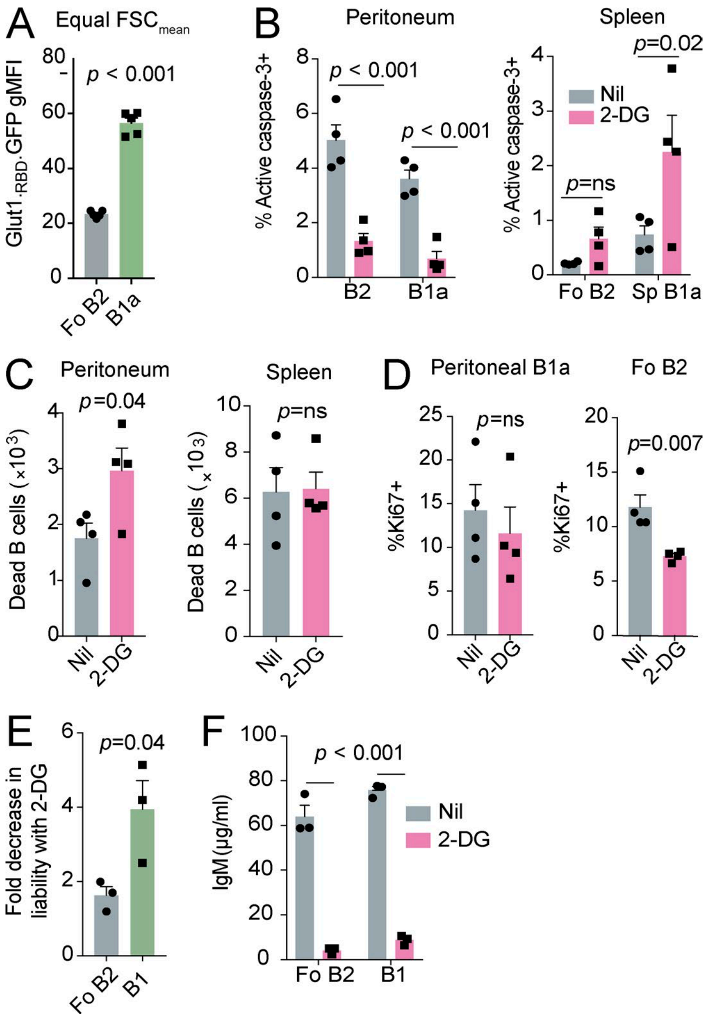

Figure S1. **Glut1 expression is higher in B1a B cells than in Fo B2 B cells after adjustment for size and the effect of 2-DG on B cell apoptosis, cell death, and IgM secretion. (A)** Glut1 expression is higher in B1a B cells than in Fo B2 B cells after adjustment for size. gMFI of Glut1.$_{RBD}$.GFP staining in B1a and B2 cells from the spleen and peritoneum gated so that each population has the same mean flow cytometric forward scatter (FSC). Each point represents one C57BL/6 mouse. Unpaired Student's *t* test used. Data from Fig. 2 A. **(B)** Effect of 2-DG on B cell death in vivo. WT C57BL/6 mice were treated with 2-DG as in Fig. 2 H. Cell death was defined as failure to exclude viability dye, assessed by flow cytometry of cells extracted from the indicated location. **(C)** Effect of 2-DG on B cell apoptosis in vivo. WT C57BL/6 mice were treated with 2-DG as in Fig. 2 H. Apoptosis was assessed by intracellular staining for active caspase-3, assessed by flow cytometry of cells extracted from the indicated location. **(D)** Effect of 2-DG on B cell proliferation in vivo. WT C57BL/6 mice were treated with 2-DG as in Fig. 2 H. Cell proliferation was assessed by intracellular staining for Ki67 and assessed by flow cytometry of cells extracted from the indicated location. (B–D) Each point represents one mouse. Unpaired Student's *t* test used. Representative of two independent experiments. **(E)** Effect of 2-DG on B cell viability in culture. Peritoneal B1 ($CD19^+CD23^-$) and splenic Fo B2 B cells ($CD19^+CD23^+$) were isolated by flow cytometry and cultured in the presence or absence of 2.5 mM 2-DG for 24 h after stimulation by 0.5 µM ODN1826. Data are presented as fold decrease in viability with treatment, as assessed by measurement of exclusion of viability dye by flow cytometry. Each point represents cells isolated from a pool of five C57BL/6 mice. Unpaired Student's *t* test used. Representative of two independent experiments. **(F)** Effect of 2-DG on IgM secretion in culture. IgM production was assessed by ELISA of supernatant from E. Two-way ANOVA with Sidak correction for multiple comparisons used. Representative of two independent experiments. ns, not significant. Mean ± SEM is depicted.

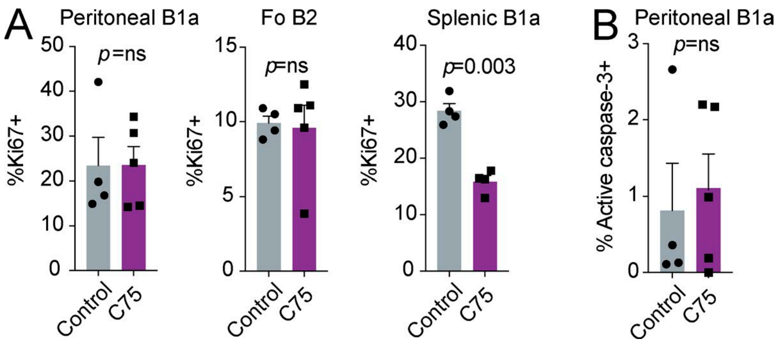

Figure S2. **The effect of C75 on B cell apoptosis and proliferation. (A)** Effect of C75 on B cell proliferation in vivo. WT C57BL/6 mice were treated with C75 as in Fig. 3 (J and K). Cell proliferation was assessed by intracellular staining for Ki67, assessed by flow cytometry of cells extracted from the indicated location. **(B)** Effect of C75 on B cell apoptosis in vivo. WT C57BL/6 mice were treated with C75 as in Fig. 3 (J and K). Apoptosis was assessed by intracellular staining for active caspase-3, assessed by flow cytometry of cells extracted from the indicated location. (A and B) Each point represents one mouse. Unpaired Student's t test used. Representative of two independent experiments. ns, not significant. Mean ± SEM is depicted.

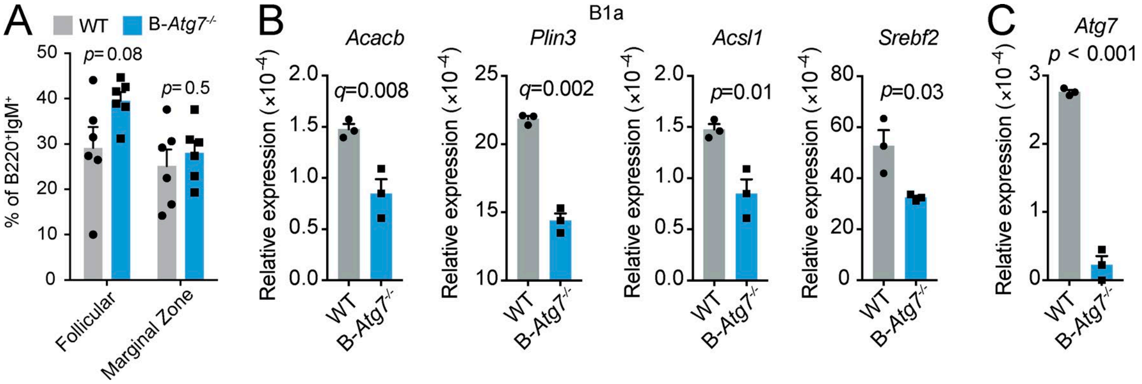

Figure S3. **B2 B cell populations in B-*Atg7*$^{-/-}$ mice, the effect of *Atg7* deletion on selected metabolic gene expression, and confirmation of efficient Cre recombinase–mediated deletion of *Atg7* in B1a B cells. (A)** Follicular and marginal B cell populations are intact in B-*Atg7*$^{-/-}$ mice. Percentages of splenic Fo B2 B cells ($CD23^+$) and marginal zone B cells ($CD23^{lo/-}CD21^{hi}$) in the $B220^+IgM^+$ population. Each point represents one mouse. Results pooled from two independent experiments. Two-way ANOVA with Sidak correction for multiple testing used. **(B)** Loss of autophagy affects metabolic gene expression. Quantitative RT-PCR gene expression data for peritoneal B1a B cells from control and B-*Atg7*$^{-/-}$ mice from experiment presented in Fig. 5 A. Data are relative to *β-actin*. P-values for *Acacb* and *Plin3* are adjusted for multiple testing using FDR method (q-value; 5% threshold). The post-hoc p-values for *Acsl*, *Srebf2*, and *Atg7* are presented unadjusted. **(C)** Mb1-cre efficiently deletes *Atg7* in peritoneal B1a B cells. Deletion efficiency of Mb1-cre for *Atg7*$^{F/F}$ in B1a B cells in control and B-*Atg7*$^{-/-}$ mice from experiment presented in Fig. 5 A. Data are relative to *β-actin*. (B and C) Each data point is the mean of two technical replicates, representing an individual mouse. Unpaired Student's t test used. Mean ± SEM is depicted.

Table S1. **List of primers used in Biomark experiments and other qPCRs**

Taqman ID	Gene
Mm00487804_m1	*Myc*
Mm00495359_m1	*Lipe*
Mm00498820_m1	*Lipa*
Mm00499536_m1	*Dgat2*
Mm00503040_m1	*Pnpla2*
Mm00507463_m1	*Acat1*
Mm01208835_m1	*Ppargc1a*
Mm01300401_m1	*Nr4a1*
Mm04208646_g1	*Plin3*
Mm01204671_m1	*Acacb*
Mm01304257_m1	*Acaca*
Mm00441480_m1	*Slc2a1*
Mm00443385_m1	*Hk2*
Mm00468869_m1	*Hif1a*
Mm01306292_m1	*Srebf2*
Mm01612132_g1	*Ldha*
Mm00439344_m1	*Hk1*
Mm01231183_m1	*Cpt1a*
Mm01309576_m1	*Pfkm*
Mm00487191_g1	*Cpt1b*
Mm00484217_m1	*Acsl1*
Mm01267402_m1	*Ldhb*
Mm01302282_m1	*Acly*
Mm00507463_m1	*Acat1*
Mm99999915_g1	*Gapdh*
Mm00436612_g1	*Slc2a4*
Mm02619580_g1	*Actb*
Mm00435587_m1	*Pfkl*
Mm03024075_m1	*Hprt*
Mm00437762_m1	*B2m*
Mm00515643_m1	*Dgat1*
Mm02525934_g1	*Ubc*
Mm01220017_m1	*Slc27a3*
